# One opioid user saving another: the first study of an opioid overdose-reversal and naloxone distribution program addressing hard-to-reach drug scenes in Denmark

**DOI:** 10.1186/s12954-019-0328-0

**Published:** 2019-12-05

**Authors:** Birgitte Thylstrup, Morten Hesse, Marian Jørgensen, Henrik Thiesen

**Affiliations:** 1 Centre for Alcohol and Drug Research, Bartholins Allé 10, 8000 Aarhus C, Denmark; 2 HealthTeam for Homeless, Save Lives Program, Sundholmsvej 18, 2300 København S, Denmark

**Keywords:** Overdose, Prevention programs, Naloxone, User, Drug scenes, Low-threshold facilities

## Abstract

**Background:**

Overdose education and naloxone distribution programs decrease opioid overdose deaths. However, no studies of such programs have been carried out in Denmark. The aim of this study was to evaluate the feasibility and the effect of a broader “training-the-trainers” model in low-threshold settings after participation in the “Danish Save Lives” [SL] program.

**Methods:**

Between May 2013 and November 2015, 552 participants from four municipalities took part in the SL program. The program is built on the train-the-trainers model where a central trainer trains others (trainers), who in turn train others (helpers). Participants were 30 police officers (5%), 188 people who use opioids (34%), 23 significant others (4%), and 217 social workers (39%). Ninety-four participants could not be classified (17%). At follow-up, participants were interviewed to determine the number and outcomes of opioid overdoses. Logistic regression was used to assess predictors of treating an overdose.

**Results:**

In all, 37 (7%) participants had intervened in 45 opioid overdose events (two trainers and 35 helpers). Detailed descriptions of the overdose event were available from 32 follow-up interviews (70%). In 16 cases, the person who intervened was already present at the site when the overdose occurred, and in 17 cases, the overdose victim recovered without complications. All overdose victims survived except one. People who used opioids were more likely to have treated an overdose than other participants (adjusted odds ratio [AOR] = 8.50, *p* = 0.001), and the likelihood of treating and overdose declined over time AOR = 0.37 (0.13, 0.93), *p* = 0.034).

**Conclusions:**

Prevention programs that target people who use opioids are more likely to be effective than programs that target professionals, especially in high-risk settings that can be hard for paramedics to reach. A future goal is to explore how prevention programs can be adapted to new user groups.

**Trial registration:**

The Danish Data Protection Agency, 2015-57-0002, Aarhus University, 2016-051-000001, 184, retrospectively registered

## Background

Many countries struggle with how to deal with opioid dependence, which often lead to fatal opioid overdoses [OD] [1]. Such events may be the result of the misuse of prescription opioid drugs [2] or may occur during opioid agonist maintenance treatment [3, 4].

In Europe, the most commonly used illegal opioid is heroin, and a recent report by the European Monitoring Centre for Drugs and Drug Addiction (EMCDDA) found that synthetic opioids (methadone, buprenorphine, and fentanyl) were increasingly being misused [5].

In response to this global situation, Opioid Overdose Education and Naloxone Distribution programs [OEND] have been expanding since 1996 [6], providing training to bystanders on how to identify the symptoms of an OD, as well as how to respond to and administer naloxone [7]. Naloxone constitutes the standard of care for medical treatment of ODs and can be administered intranasally, intramuscularly, intravenously, or subcutaneously; the major adverse effect is precipitated opioid withdrawal, due to the rapid displacement of opioid agonist from the opioid receptor [8].

Over the past two decades, hundreds of OEND programs have demonstrated their feasibility, reporting decreases in OD mortality following implementation (e.g., [9–13]). Given a sufficiently fast dissemination of the programs, a large number of deaths can be prevented in the event of a significant epidemic [13, 14]. There is emerging evidence that the OEND programs can be expanded among people who use opioids (PWUO) (e.g., [15–18]), clinicians [19], police officers [20], and significant others, such as family members [21].

Naloxone distribution programs have been established in a growing number of countries, e.g., Scotland [22, 23] and Wales [16], and the distribution of naloxone is legalized in countries such as Norway [24], Sweden [25], and England [26]. In Denmark, the distribution of naloxone is allowed, however, despite considerable attention towards the issue of overdoses, such as substitution treatment programs, injection rooms, and heroin clinics [27, 28], Denmark still ranks among those countries in Europe with the highest rate of annual OD deaths [29, 30], with a rate of more than > 40 per million in the adult population [31].

The high prevalence of OD deaths in Denmark has received much attention, however, only a few studies have investigated potential causes. The largest and most recent of these studies investigated drug-induced and other drug-related deaths in Denmark between 2008 and 2011. Findings from the study stressed the need to increase OEND program involvement to PWUO who are often hard to reach for more traditional substance abuse treatment settings. These include PWUO in residential areas and in private residences with less access to professional help in case of an OD, as well as PWUO who are not in contact with the public health system and treatment system in general [32]. Also, the study drew attention to the high rates of OD deaths for people who had been receiving methadone maintenance treatment for more than 1 year at the time of death [33].

In response to the high numbers of OD incidences in Denmark, the Danish “Save Lives” [SL] program was initiated by the *HealthTeam for Homeless in 2010*. The aim of the SL program was to support the reduction of both fatal and non-fatal OD by offering OD education and naloxone. One specific aim of the SL was to reach PWUO, who are often difficult to access by the Danish public health and treatment system. We therefore aimed to provide training in low-threshold facilities, i.e., public service programs that make minimal demands on the patient and offer services without attempting to control their intake of drugs, only providing counseling if requested [34].

To develop a locally embedded SL program, the “train-the-trainers” model (TTT) was used. In this model, a central trainer trains others, who then in turn train others within a specific target population [35]. The TTT model has been used to address different target groups [36] and shows potential in an e-learning format [37]. An advantage to this model is that it has the potential to build capacity to maintain competency to intervene in case of an opioid OD at a local level, where the trainers are often already working with the target group and thus have more direct access to carry out the intervention. A further advantage is that the TTT enables training a large number of potential bystanders (helpers) in responding to an OD in a relatively short time and at a relatively low cost.

The present study evaluated the feasibility and effect of the SL program in Denmark. Our aim was to assess the use of the TTT model in low-threshold settings and to determine whether the approach would increase access to treating ODs in settings that are otherwise difficult for relevant professionals to reach.

## Methods

### Setting

From April 2013 until the end of May 2015, SL workshops for trainers were held at various low-threshold facilities in four Danish municipalities which had high rates of ODs. The municipalities were the three largest cities in Denmark, Copenhagen (city population 775,033), Aarhus (city population 261,570), and Odense (city population 178,210), and Glostrup, a suburb of Copenhagen (population 22,663).

### Participants

The SL project coordinator (a registered nurse) invited the management of community substance abuse treatment institutions, methadone and heroin maintenance clinics, homeless shelters, drop-in centers, and police in the four municipalities to participate in the SL trainer workshops. Also, PWUO affiliated with the Danish Drug Users Union in Copenhagen were invited. The management at each site determined the number of participants, and participation was independent of educational background, voluntary, and held no additional benefits besides the SL training.

Following the training workshop, trainers invited other staff at their work, PWUO, and significant others (family members, partners, and friends who knew a person who used opioids) to participate in the helper workshops. Invitations were extended both on a one-to-one basis, as a public announcement, and as part of a collaboration with other institutions. Information on those who did not choose to attend the trainer and helper workshops was not collected.

### The SL program and procedures

The training material for the SL program was adapted from a similar material used at the Boston Health Care Team for the Homeless, kindly provided to us by Dr. Jessica Gaeta.

The trainer workshops lasted 4 h and covered: [1] background and rationale for the SL program, [2] mechanisms of an OD, [3] identifying and responding to an OD, [4] effects of naloxone, [5] assembling the naloxone kit and administrating the naloxone, [6] pulmonary resuscitation [PR] using a standard reanimation “AmbuMan” training mannequin, [7] project documentation, and [8] implementation procedures at the local sites.

The OD prevention kit included one box with Prenoxad (5 doses of 0.4 mg), one MAD300 nasal atomizer, one ventilation mask, cotton, and surgical alcohol wipes, gloves, a card with name, and MD signature for dispensing naloxone, a card with project information, a pencil, and a registration sheet. Participants were instructed to deliver 0.4 ml (0.4 mg) in each nostril of the OD victim, ventilate, and treat again if OD symptoms did not subside within 2 min. The only available naloxone in glass ampoules (0.4 mg/ml) was used, but due to problems with use, the study changed to Prenoxad when it was released in the UK in 2012. The Prenoxad has a 2.5 times higher concentration of naloxone (1 mg/ml), and comes in prefilled syringes, which reduces the risk of cuts and infections when treating ODs under stressful conditions.

Participants at the trainer workshops completed a test on how to treat and teach an OD response with naloxone. In case of incorrect test answers, the test and the understanding of the skills required to become an SL trainer would be re-examined. Following this, the trainers received a material for teaching the helper workshops, as well as instructions on how to refill and register the naloxone distribution to the helpers at their local site. At each workshop, a local medical doctor (MD) managed the dispensing of naloxone to each workshop participant, using a standardized report form for documenting dispensations. Further, the SL project coordinator (a registered nurse) supervised the trainers when they conducted their first helper workshop. The helper workshop lasted 1 h, involved 5–6 participants, and covered [1] participant experience with ODs and risk behavior [2]; introduction to the naloxone kit and intranasal spray [3]; resuscitation training.

Participants at both the trainer and helper workshops completed a one-page questionnaire with information which included questions about gender, age, history of opioid use, experiencing/observing ODs, and whether the respondent was a PWUO, a significant other, or a professional. To support ongoing workshop activities and ensure the quality of the information provided to the trainers and helpers, the project coordinator maintained contact with all the facilities, in which the workshops had taken place. Throughout the project period, the project coordinator also kept track of the number of treated ODs and recorded who had treated an overdose and how the overdose had been treated. All the participating trainers were asked to collect and return old rescue kits in order to obtain new rescue kits, which were distributed by the project coordinator.

Furthermore, from February through November of 2015, the project coordinator conducted follow-up interviews with both the trainers and helpers in order to validate the number and outcomes of the reported OD reversals. The follow-up interviews were conducted in person and over the phone, depending on how the individual study participant was most easily reached. The return of kits and the follow-up contact were the two primary sources of information on both the number and procedures of the treated OD incidents during the project period.

Participants who were unable to follow the instructions and to demonstrate the procedures of resuscitating an overdose victim were not given naloxone, and no record was made of them, including their gender or age.

### Statistical analysis

The likelihood of having treated an OD during the study period was assessed using logistic regression. As predictors, we used gender, age, the year of the training course, and whether the respondent was a PWUO. Year and age were both entered as continuous predictors. We defined PWUO as those who indicated that they were currently receiving substitution treatment, as well as those who indicated that they had used opioids to get intoxicated in the past, or those who indicated both.

Follow-up rates were compared across groups in order to assess whether differential attrition affected results. Random effects were specified for the day of training nested within the town in which training took place in order to control for class effects. The date of the course was added, as participants who received training earlier had more time to treat overdoses. All statistical analyses were conducted using Stata 15 IC for Windows [38].

### Ethics

The current implementation study was put out to tender by the Danish Board of Health with the aim of investigating the potential for a broader implementation of an OEND program project in Denmark. The study was deemed exempt from a formal evaluation, and the Danish Board of Health was involved in the ethical guidelines of the project. The project was conducted in accordance with the Declaration of Helsinki 2004, which states that it is the duty of the researcher to protect the life, health, dignity, integrity, right to self-determination, and privacy and confidentiality of personal information of research subjects [39]. All participants were informed about the nature of the study and told that they were free to withdraw from the study at any time and that participation would not interfere with any treatment or services with the facilities that they were in contact with.

The Danish Medicines Agency (Lægemiddelstyrelsen) gave permission to use of Prenoxad (Lægemiddelstyrelsen 2013: Approval # 2013071299; Lægemiddelstyrelsen 2014: Approval # 2014093655; Lægemiddelstyrelsen 2016: Approval # 2016013522).

## Results

### Participants

During the study period, the SL program provided workshops for 552 participants (188 PWUO, 34%; 217 social workers, 39%; 30 police officers, 5%; 23 significant others, 4%; and 94 others who could not be classified due to missing data, 17%; see Table 1). Of the 552 participants, 262, 47% were women (25% of the PWUO and 59% in the remaining sample), and the mean age was 42 years (standard deviation [SD] = 11, range 20 to 87, 44 for the PWUO and 40 for the remaining participants). All of the PWUO had previously or were currently using opioids, 55% of the PWUO had previously experienced an OD. Of the PWUO, 88% had previously witnessed at least one OD compared to 50% in the remaining sample.

**Table 1:** Frequency distribution of selected sample characteristics

	Police officers (*n* = 30)	PWUO (*n* = 188)	Significant others (*n* = 23)	Social workers (*n* = 188)	Unclassified (*n* = 94)	Total (*n* = 552)	Statistic
Female gender	7%	25%	74%	66%	44	74%	*Χ*^2^ (4) = 97.10
Age (median IQR)^a^	36 (33–39)	45 (39–50.5)	48 (40–54)	38 (30–47)	44 (30–55)	41 (33–50)	*Χ*^2^ (4) = 36.83
Living alone^b^	13%	74%	64%	25%	37%	45%	*Χ*^2^ (4) = 113.13
Previously witnessed OD	77%	88%	68%	52%	26%	63%	*Χ*^2^ (4) = 101.73
Previously experienced OD	0%	55%	14%	1%	0%	21%	*Χ*^2^ (4) = 210.84

^a^Five respondents did not report age

^b^Thirty-three participants did not respond to this item

### Follow-up interviews

Follow-up took place on average 358.5 days after participation in the SL workshop (SD = 219, median = 351). Follow-up data was available from 174 (32%) of the participants. Of the 174 interviewees, 77 (44%) were women. The follow-up sample was compared to the remaining trainee sample, and there were no differences in age and gender. However, the proportions of the various groups differed between the follow-up sample and the remainder of the trainee sample, with social workers having the highest participation rate (42.9%), followed by PWUO (33.0%), police officers (33.3%), and significant others (4.4%). The difference in participation was significant (*χ*^2^(4) = 44.1, *p* < 0.001).

### Treatment of ODs

In all, 37 participants or 7% of all participants had treated at least one OD after their first workshop participation (45 ODs in all). This information was confirmed through the follow-up interview or when the participant picked up a new kit. Four of the 37 persons made three OD reversals, one person made two OD reversals, and the remaining 31 made one OD reversal. In the treated OD occurrences, 65% reported that they had administered naloxone at the first overdose that they had treated, of which 57% had administered naloxone intranasally (using Prenoxad from the study), while the rest had used a kit with glass ampules which had been dispensed earlier in the pilot study. Of the 37 participants, 22 (59%) reported whether they had called the emergency services or not, and of these, 12 (55%) reported that they called the emergency services. In all, 6 of 15 (40%) PWUO and 6 of 7 (86%) social workers had called the emergency services. None of the police officers, 15% of the PWUO, none of the significant others, 4% of the social workers, and 1% of the unclassified participants had treated an OD during follow-up (see Table 2). Of those who treated an OD, two of 37 were trainers (5%), and of those who did not treat an OD, 40 of 515 were trainers (8%).

**Table 2:** Treated overdose

	Police officers (*n* = 30)	PWUO (*n* = 188)	Significant others (*n* = 23)	Social workers (*n* = 217)	Unclassified (*n* = 94)	Total (*n* = 552)	Statistic
Treated an overdose	0 (0%)	28 (15%)	0 (0%)	8 (4%)	1 (1%)	36 (6%)	*Χ*^2^ (4) = 31.91

A total of 616 of OD prevention kits were distributed during the project period, including both initial kits and refills.

### Predictors of trainers/helpers who treated who treated an OD

The results of the logistic regression analysis are summarized in Table 3. In the unadjusted models, older age, being a PWUO, and later year of training were all associated with treating an OD. Overall, the Wald test for the multivariate model was significant (*χ*^2^(4) = 15.47, *p* < 0.001). PWUO were more likely than non-PWUO to have treated an OD (adjusted odds ratio [AOR] = 8.50 confidence interval [CI] = 3.34 to 30.94, *p* = 0.001). No other factors were significantly associated with treating an OD, but a considerable group effect was observed, indicating that individuals who had been trained in the same workshop had a similar likelihood of treating an OD (*ρ* = 0.47).

**Table 3:** Predictors of treating an overdose using logistic regression (*N* = 547)

Predictor variable	OR (95% C.I.)	*p* value	AOR (95% C.I.)	*p* value
Age	1.04 (1.01, 1.07)	0.011	1.01 (0.97, 1.06)	0.549
Male gender	1.50 (0.75, 2.97)	0.251	0.83 (0.32, 2.13)	0.694
Professionals or significant others	Reference		Reference	
PWUO	6.81 (3.14, 14.76)	0.000	8.50 (3.34, 30.94)	0.001
Year of training	0.41 (0.26, 0.66)	0.000	0.37 (0.13, 0.93)	0.034
Intra class correlation coefficient site	*ρ* < 0.01		*ρ* < 0.01	
Intra class correlation coefficient class	*ρ* = 0.49(0.26, 0.73)		*ρ* = 0.47(0.23, 0.72)	

Five participants had missing data on age and had to be excluded from this analysis

### Characteristics of people with treated ODs

Of the 45 treated ODs, follow-up data was available for 26 specific events involving 19 men overdosing and seven women, and in all but two cases naloxone was administered. In 20 of the 26 events (85%), the person who had treated the OD was already present at the site when the OD occurred, and in ten of the events, cardiopulmonary resuscitation (CPR) was given (50%). In 12 of the 26 events, an emergency call was made (46%), and in 11 of the events, where an ambulance was called, ambulance staff took over the treatment (92%). Out of the 26 events, 19 (73%) of the OD victims recovered without complications. In cases with complications, one victim drifted between states of consciousness, five had cramps and vomited, and one died. According to the individual report, the deceased was already dead at the time of naloxone dispensation, and five doses of naloxone were given intramuscularly without any response.

### Training the trainers’ effects

In terms of dissemination of the SL program, ten trainer workshops and 86 helper workshops were conducted after the initial round of trainer workshops. Of the 43 trainers, 16 (37%) did not provide any helper workshops, while the remaining 26 did (60%), and one trainer did not respond to this question (2%). Among the 16 trainers who did not conduct any helper workshops, the most commonly endorsed reason was lack of time (*n* = 12), followed by lack of interest from PWUO (*n* = 8, four indicated both of these reasons). There was no significant difference in the level of dissemination between the four municipalities where the trainer workshops had taken place. There was a significant difference between trainers who had participated in the study period and trainers who had participated at a later stage; trainers who had participated in the study at an earlier period had completed significantly more workshops.

## Discussion

This study is the first study in Denmark that evaluated the feasibility of training bystanders to perform pulmonary resuscitation and administer naloxone after participation in the SL program. The study adds to the growing evidence that OD education and naloxone distribution can be delivered. We found that OD resuscitation and naloxone administration were delivered successfully in all but one of the reported OD incidents. In this case, the individual report stated that the person was already deceased before the naloxone was administered. We also found that PWUO reported the highest rate of interventions and that especially older PWUO reported the administration of naloxone.

In contrast to some studies, we found that police officers and significant others reported complete absence of OD interventions during the observation period and that PWUO were more likely to call emergency services compared to professionals [40, 41]. These findings are important, as they suggest that further effort is still required in order to increase the involvement of police officers and significant others in the prevention of OD. Police officers and family members are among the most likely people to be nearby when ODs take place in public spaces and in social settings. The finding that PWUO in this study were more likely to call emergency services supports the positive role that service users may play in reversing ODs. This finding also indicates that prevention programs have the ability to support hard-to-reach PWUO in acting as public health collaborators, rather than being exclusively victims [42]. A growing body of research supports that harm reduction interventions are not divorced of the types of social exchange and interpersonal relationships that surround PWUO and that peer-based harm reduction is important for facilitating improved health and wellbeing of PWUO within their own communities [43], where people who use or have used drugs are a key component [44].

The TTT model was successful as it led to a large number of helper workshops. However, 38% of the trainers did not provide any helper workshops, emphasizing that a closer follow-up on implementation procedures following the trainer workshops may be needed. Such a follow-up procedure could include more involvement of the management at the participating sites in order to ensure adequate allocation of resources and support staff interest and commitment to the SL program [45]. Studies in other fields have shown similar challenges regarding the replication of training sessions at the local level; a study found that only 20% of those trained in disaster preparedness conducted a replication training 6 months after the training [46], and a study on perinatal HIV prevention and care found that only 20% training went on to conduct training after being trained [47].

Only 8% of the distributed naloxone kits were used to reverse an OD by only 7% of the SL participants during the project period. This is significantly less compared to some other studies [23, 48]. Despite the rather low use of reversal kits, we believe that this study demonstrates that OEND programs are not only feasible in traditional drug treatment settings, but can make a difference in settings that encounter PWUO who often are more difficult to reach [32]. We also believe that the present study shows the need for continuous support when implementing OEND prevention programs in relation to both traditional treatment settings and low-threshold settings in Denmark, reaching PWUO at risk of experiencing or witnessing an opioid OD.

Despite the low distribution and use of kits, this study suggests that even brief opioid OD prevention programs are able to save lives at a considerably low cost (e.g., [49–51]). A study by Coffin et al. found that naloxone distribution to heroin users was cost-effective, even under markedly conservative assumptions, where one death was prevented for every 227 naloxone kits distributed. A recent estimation found that community-based naloxone distribution is not only beneficial for PWUO, but also cost-effective, and that service programs that also address the underlying substance use disorder or reduce transmission of HIV and hepatitis C virus (HCV) may be even more cost-effective when compared with no additional intervention [52].

Opioids still constitute the main cause of drug-related deaths, and mortality that is caused by prescription opioids now challenges heroin as the main agent in fatal drug ODs. Thus, it is important to continue the development of OEND in order to reach new target groups, such as individuals who are prescribed more than 100 mg per day of oral morphine equivalents who have a previous history of OD, who have recently been released from prison or discharged from residential treatment, or who use alcohol, benzodiazepines, or other drugs in combination with opioids [53].

Recent studies point towards the similarity of trends in ODs caused by prescription opioids in the Nordic countries [30, 54] compared to trends in Europe [55], and especially Canada [56] and the USA [57]. Furthermore, there is growing evidence that fentanyl potentially increases the risk of severe and even fatal poisoning [5]. In such circumstances, dispensing naloxone and training bystanders in correct naloxone administration need to be re-assessed in order to reach new target groups [58]. This may well be the case for Denmark, where there seems to be an increase in deaths associated with fentanyl analogs with ten deaths being reported to the National Health Authority in 2018 [59]. To meet these challenges, future prevention strategies in Denmark should develop programs for new user groups, which would involve an early scale up of OEND programs [14], possibly using new channels of communication [60, 61]. Of interest here is a recent study from Australia that found that community pharmacists were willing to supply naloxone, but expressed a need for training in how to identify the symptoms of an OD and how to respond and to administer naloxone [62].

A perspective that is worth considering is whether the availability of antidotes in the hands of laypersons can diminish the perception of public responsibility for drug overdoses. It may be that increased availability of antidotes among laypersons may entrench the existing health inequity between marginalized people and the general population by resulting in access to only minimal healthcare to marginalized populations in the form of the most acute services, compared to access to high-quality healthcare for the general population [63]. To support the development of OEND programs and health care to marginalized people in general, future prevention strategies should also involve the focus on the consequences of treating illegal drug use primarily as a criminal activity and only secondarily as a public health concern. Although supervised injection sites and safer smoking rooms may not substantially reduce the number of PWUO and other injection drugs, they have the potential to attenuate the serious medical sequelae of opioid use by facilitating discussions about how to reduce negative consequences of use [64, 65]. Also, it is likely that while becoming an OD responder may involve taking up a new positive social role, it may also involve additional stress that may require additional access to professional support, in order to cope successfully [42, 66]. Although intranasal naloxone, even with suboptimal concentrations, has shown an effect on opioid ODs [17], new devices developed in the US dispense naloxone intranasally in amounts and concentrations that will ensure a proper dosage under all resuscitation circumstances.

Our study has several limitations. The interpretation of the impact of the SL program is limited due to lack of comparison or control groups, the high proportion of missing data on the type of participant, and the low follow-up rate. Also, we were not able to validate reports of OD through police and ambulance service records. It is likely that some participants who responded to an OD did not return their kit or responded to the follow-up and that the number of ODs reported in this study therefore is lower than the overdoses actually treated. Furthermore, we employed a short questionnaire in this study, which omitted a detailed description of the participants. We found this approach to be necessary in order to reach the PWUO in the study, some of whom had very short attention spans. At the time of this writing, records of the individual workshops had been deleted, and it was not possible for us to describe the total number of each type of workshop that had been conducted. Finally, the specific opioids involved in the treated ODs were not recorded which has prevented us from reporting the proportional contribution of specific opioids (such as heroin), as well other causal agents (such as alcohol or benzodiazepines) in the OD episodes.

## Conclusion

Reducing drug-related deaths remains a major challenge for public health policy globally and in Denmark. This study shows that countermeasures to opioid ODs require multidisciplinary responses, especially in high-risk settings, which are often difficult for paramedics to reach. An important future aim is to explore how prevention programs can be adapted to new user groups.

## Data Availability

The data are stored at the Centre for Alcohol and Drug Research, Aarhus University, in accordance with rules and regulations for data storage. The data are available from the corresponding author on a reasonable request.

## Abbreviations

HIV: Human immunodeficiency virus
OD: Opioid overdoses
OEND: Opioid Overdose Education and Naloxone Distribution Programs
PWUO: People who use opioids
SL: Save Lives Program
